# Impact of time to distant recurrence on breast cancer-specific mortality in hormone receptor-positive breast cancer

**DOI:** 10.1007/s10552-022-01561-2

**Published:** 2022-02-28

**Authors:** Gregory S. Calip, Nadia A. Nabulsi, Colin Hubbard, Alemseged A. Asfaw, Inyoung Lee, Jifang Zhou, Jenilee Cueto, Debanjali Mitra, Naomi Y. Ko, Kent F. Hoskins, Ernest H. Law

**Affiliations:** 1 Department of Pharmacy Systems, Outcomes and Policy, University of Illinois at Chicago, Chicago, IL US; 2 Patient & Health Impact, Pfizer, Inc, New York, NY US; 3 School of Medicine, Section of Hematology Oncology, Boston University, Boston, MA US; 4 Division of Hematology and Oncology, University of Illinois at Chicago, Chicago, IL US; 5 Center for Pharmacoepidemiology and Pharmacoeconomic Research, University of Illinois at Chicago, 833 South Wood Street MC 871, Chicago, IL 60612 US

**Keywords:** Distant recurrence-free interval, Early-stage BC, Mortality, Metastatic BC

## Abstract

Women with hormone receptor (HR)-positive early-stage breast cancer (BC) have five-year survival rates of > 90% but remain at serious risk for developing distant metastases beyond five years from diagnosis. This retrospective cohort study used data from the Surveillance, Epidemiology, and End Results (SEER) registries to examine associations between distant recurrence-free interval (DRFI) and risk of BC-specific mortality following distant relapse. The analysis includes 1,057 women with second primary stage IV BC who were initially diagnosed with AJCC stages I–III HR-positive BC between1990 and 2016. Overall, 65% of women had a preceding DRFI of ≥ 5 years. Five-year BC-specific survival following development of distant recurrence was 52% for women with DRFI ≥ 5 years compared to 31% in women with DRFI of < 5 years. In multivariable analyses, risks of cancer-specific mortality following distant recurrence were lower in women with DRFI of 5 years or more (subdistribution hazard ratio = 0.72, 95% CI 0.58–0.89, *p* = 0.002). The results of this study may inform patient-clinician discussions surrounding prognosis and treatment selection among HR-positive patients who develop a distant recurrence of disease.

**Supplementary Information:**

The online version contains supplementary material available at 10.1007/s10552-022-01561-2.

## Introduction

More than 150,000 women in the USA live with metastatic breast cancer (BC). While some patients presenting with invasive BC are diagnosed with de novo metastatic disease, > 90% first present with locoregional (stage I to III) disease [1, 2]. Women with hormone receptor HR-positive (HR +) BC that is confined to the breast and ipsilateral axillary lymph nodes generally have a favorable short-term prognosis with five-year relative survival rates of 98%, 94%, and 82% for American Joint Committee on Cancer (AJCC) stages I, II, and III disease, respectively [2]. However, the risk of distant recurrence persists for decades, with up to 23% and 38% of patients with HR + disease experiencing distant recurrence by five and 25 years after diagnosis, respectively [3].

A goal of adjuvant therapies is to prevent recurrence, but even women who eventually relapse may derive benefit from prior systemic adjuvant treatment as it may delay the time to recurrence, providing downstream benefit in survival outcomes [4]. No studies to date have evaluated whether prolonging the distant recurrence-free interval (DRFI) in the largest BC subtype (HR + disease) is associated with improved survival following the development of recurrent distant metastases. This study evaluated the association between DRFI and the risk of BC-specific mortality among women with recurrent metastatic HR + BC.

## Methods

A retrospective cohort study was conducted using the Surveillance, Epidemiology, and End Results Program SEER*Stat database [5]. Between 1990 and 2016, 1,651 women aged 18 years and older were identified with stage IV HR + BC, coded as a second BC malignancy. Among this group, 1,057 had a prior first primary HR + , stage I–III BC documented in SEER. Patient and tumor characteristics were collected for both first and second BC diagnoses, including age and year of diagnosis, race, marital status, AJCC stage, tumor grade and size, nodal status, surgery type, radiotherapy, and chemotherapy status. For metastatic BC women, information on site(s) of metastasis was available in 2010 and later.

### Exposure and outcomes

The primary exposure of interest was DRFI (≥ 5 years vs. < 5 years), calculated as the time from first primary stage I–III HR + BC diagnosis until second HR + metastatic diagnosis [5]. The outcome of interest, BC-specific mortality, was collected from SEER registry records with valid months of follow-up and vital status information. Women without post-diagnosis information were excluded. Overall and non-BC mortality data was also collected to account for competing risks of death.

### Statistical analyses

For time-to-event analyses, women were followed in months since first and second BC diagnoses until death or the end of the study period (December 2017). Incidence of BC-specific mortality was estimated using cumulative incidence functions [6] from competing risks regression models by length of DRFI and equality of the functions was determined using Gray’s test [7, 8]. Multivariable Fine and Gray competing risks regression models were used to estimate subdistribution hazard ratios (SHR) and 95% confidence intervals (CI) associated with length of DRFI, accounting for death to causes other than BC as a competing risk [9]. Women contributed at-risk time for BC-specific mortality in the model beginning at the month of their stage IV diagnosis. Multivariable models were adjusted for age at metastatic diagnosis (< 45, 45–54, 55–64, 65–74, 75 + years), year of metastatic diagnosis (1990–1999, 2000–2009, 2010–2016), grade of stage IV BC (1, 2, 3–4), chemotherapy for metastatic disease (yes, no/unknown), radiation for metastatic disease (yes, no/unknown), and metastatic sites involved at stage IV diagnosis (yes, no, unknown). Separately, in sensitivity analyses, we varied our primary approach by examining DRFI intervals of < 2, 2–5, 5–7, 7–10, and 10 + years.

All tests were two-sided and *p* values < 0.05 were considered statistically significant. All statistical analyses were performed using Stata/MP version 16.1 (College Station, TX: StataCorp LLC).

## Results

Of 1,057 women with recurrent metastatic HR + BC eligible for analysis, 369 (35%) were diagnosed with metastatic BC within 5 years after the first primary BC. The median age at first primary BC diagnosis was 54 (IQR 44–64) and most women were first diagnosed at AJCC stages I or II (72%) (Table 1). Compared to women with DRFI of < 5 years, women with DRFI of ≥ 5 years had first primary tumors that were smaller (< 2 cm, 59 vs. 35%) and a smaller proportion were node-positive (50 vs. 69%).

**Table 1:** Characteristics of women diagnosed with stages I-III hormone receptor-positive breast cancer by subsequent distant recurrence-free interval

	All women (*n* = 1,057)		DRFI < 5 years (*n* = 369)		DRFI ≥ 5 years (*n* = 688)	
	*n*	(%)	*n*	(%)	*n*	(%)
Age at primary BC diagnosis, years						
Mean (SD)	54.4	(13.1)	55.1	(14.0)	54.0	(12.6)
Median (IQR)	54	(44–64)	55	(44–65)	53	(44–63)
< 45	267	(25.3)	95	(25.7)	173	(25.1)
45–54	295	(27.9)	86	(23.3)	209	(30.4)
55–64	242	(22.9)	96	(26.0)	146	(21.2)
65–74	173	(16.4)	58	(15.7)	115	(16.7)
75 +	80	(7.6)	35	(9.5)	45	(6.5)
Primary BC diagnosis year						
1990–1999	320	(30.3)	74	(20.1)	246	(35.8)
2000–2009	624	(59.0)	197	(53.4)	427	(62.1)
2010–2016	113	(10.7)	98	(26.6)	15	(2.2)
Race						
White	781	(73.9)	257	(69.6)	524	(76.2)
Black	171	(16.2)	72	(19.5)	99	(14.4)
Other	104	(9.8)	40	(10.8)	64	(9.3)
Marital status						
Unmarried	442	(41.8)	168	(45.5)	274	(39.8)
Married	583	(55.2)	190	(51.5)	393	(57.1)
Stage						
I	356	(33.7)	85	(23.0)	271	(39.4)
II	407	(38.5)	120	(32.5)	287	(41.7)
III	294	(27.8)	164	(44.4)	130	(18.9)
Grade						
1	143	(13.5)	39	(10.6)	104	(15.1)
2	445	(42.1)	148	(40.1)	297	(43.2)
3 and 4	365	(34.5)	147	(39.8)	218	(31.7)
Unknown	104		35		69	
Tumor size, cm						
< 2	533	(50.4)	130	(35.2)	403	(58.6)
2–5	360	(34.1)	136	(36.9)	224	(32.6)
> 5	161	(15.2)	103	(27.9)	58	(8.4)
Nodal status						
Negative	460	(43.5)	113	(30.6)	347	(50.4)
Positive	597	(56.5)	256	(69.4)	341	(49.6)
1 to 3	267	(25.3)	82	(22.2)	185	(26.9)
4 +	330	(31.2)	174	(47.2)	156	(22.7)
Laterality of primary BC						
Right	550	(52.0)	180	(48.8)	370	(53.8)
Left	507	(48.0)	189	(51.2)	318	(46.2)
Surgery						
Breast-conserving	565	(53.5)	141	(38.2)	424	(61.6)
Mastectomy	459	(43.4)	205	(55.6)	254	(36.9)
Unknown type	33		23		10	
Radiation						
None/unknown	476	(45.0)	178	(48.2)	298	(43.3)
Any	557	(52.7)	180	(48.8)	377	(54.8)
Chemotherapy						
None/unknown	526	(49.8)	160	(43.4)	366	(53.2)
Any	531	(50.2)	209	(56.6)	322	(46.8)
*Characteristics at metastatic diagnosis*						
Age at metastatic diagnosis, years						
Mean (SD)	61.8	(13.3)	57.5	(13.8)	64.2	(12.5)
Median (IQR)	62	(52–72)	57	(46–67)	63	(55–74)
< 45	115	(10.9)	79	(21.4)	36	(5.2)
45–54	206	(19.5)	74	(20.1)	132	(19.2)
55–64	296	(28.0)	104	(28.2)	192	(27.9)
65–74	223	(21.1)	59	(16.0)	164	(23.8)
75 +	217	(20.5)	53	(14.4)	164	(23.8)
mBC diagnosis year						
1990–1999	67	(6.3)	53	(14.4)	14	(2.0)
2000–2009	341	(32.3)	161	(43.6)	180	(26.2)
2010–2016	649	(61.4)	155	(42.0)	494	(71.8)
mBC grade						
1	90	(8.5)	29	(7.9)	61	(8.9)
2	389	(36.8)	127	(34.4)	262	(38.1)
3 and 4	338	(32.0)	143	(38.8)	195	(28.3)
Unknown	240	(22.7)	70	(19.0)	170	(24.7)
Radiation for mBC						
None/unknown	476	(45.0)	280	(75.9)	526	(76.5)
Any	557	(52.7)	84	(22.8)	152	(22.1)
Chemotherapy for mBC						
None/unknown	526	(49.8)	180	(48.8)	387	(56.3)
Any	531	(50.2)	189	(51.2)	301	(43.8)
*Metastatic site involvement (2010* +*)*	*n* = 649		*n* = 155		*n* = 494	
Bone						
No	209	(32.2)	48	(31.0)	161	(32.6)
Yes	420	(64.7)	98	(63.2)	322	(65.2)
Unknown	20		9		11	
Brain						
No	569	(87.7)	129	(83.2)	440	(89.1)
Yes	47	(7.2)	15	(9.7)	32	(6.5)
Unknown	33		11		22	
Liver						
No	492	(75.8)	104	(83.2)	388	(89.1)
Yes	127	(19.6)	41	(26.5)	86	(17.4)
Unknown	30		10		20	
Lung						
No	424	(65.3)	111	(71.6)	313	(63.4)
Yes	192	(29.6)	34	(21.9)	158	(32.0)
Unknown	33		10		23	

*DRFI* distant recurrence-free interval, *IQR* interquartile range, *mBC* metastatic breast cancer, *SD* standard deviation

The median age at diagnosis of metastatic BC was 62 years (IQR 52–72); women with DRFI of < 5 years were younger compared to women with DRFI of ≥ 5 years (57 vs. 63 years) (Table 1). Women with DRFI < 5 years were more likely to be diagnosed with higher grade metastatic disease (grades 3–4, 39 vs. 28%) and receive subsequent chemotherapy (51 vs. 44%), but had similar rates of radiotherapy for metastatic BC (23 vs. 22%). Among women with information on site of metastatic involvement, those with DRFI of ≥ 5 years had greater involvement of liver metastases (27 vs 17%) but less lung metastases (22 vs 32%) compared to women with DRFI of < 5 years.

Women with DRFI < 5 years had a five-year BC-specific survival rate of 31% compared to 52% in women with DFRI of ≥ 5 years. Cumulative incidence functions for BC-specific mortality indicated that women with DRFI of < 5 years had significantly higher incidence of BC mortality after accounting for competing risks (*p* = 0.026). Women with DRFI of < 5 years had a slightly greater incidence of other-cause death (8 vs. 5%) by the end of the follow-up period. In multivariable Fine and Gray models adjusted for age, diagnosis year, metastatic BC grade, receipt of radiation, receipt of any chemotherapy and metastatic site involvement, women with DRFI of ≥ 5 years had a lower risk of BC-specific mortality compared to women with DRFI of < 5 years (SHR 0.72, 95% CI 0.58–0.89, *p* = 0.002) (Table 2).

**Table 2:** Results from Fine and Gray competing risks regression models reporting subdistribution hazard ratios and robust 95% confidence intervals for risk of breast cancer-specific mortality following distant recurrence of hormone receptor-positive breast cancer

	Crude SHR	95% CI	*p*	Adjusted SHR	95% CI	*p*
DRFI						
< 5 years	1.00	Reference		1.00	Reference	
≥ 5 years	0.67	(0.57, 0.79)	< 0.001	0.72	(0.58, 0.89)	0.002
Age at mBC diagnosis						
< 45	1.00	Reference		1.00	Reference	
45–54	0.70	(0.54, 0.90)	0.005	0.90	(0.66, 1.22)	0.498
55–64	0.82	(0.65, 1.03)	0.085	1.03	(0.78, 1.36)	0.834
65–74	0.71	(0.55, 0.92)	0.009	0.91	(0.67, 1.25)	0.581
75 +	1.01	(0.78, 1.32)	0.926	1.52	(1.07, 2.16)	0.019
mBC diagnosis year						
1990–1999	1.00	Reference		1.00	Reference	
2000–2009	0.68	(0.51, 0.90)	0.008	0.77	(0.53, 1.11)	0.157
2010–2016	0.53	(0.40, 0.71)	< 0.001	0.43	(0.27, 0.68)	< 0.001
Race						
White	1.00	Reference		1.00	Reference	
Black	1.28	(1.04, 1.58)	0.020	1.23	(0.96, 1.59)	0.102
Other	1.06	(0.80, 1.40)	0.682	1.13	(0.81, 1.57)	0.472
Grade						
1	1.00	Reference		1.00	Reference	
2	1.42	(1.05, 1.94)	0.024	1.27	(0.91, 1.77)	0.156
3 and 4	1.73	(1.26, 2.37)	0.001	1.47	(1.04, 2.07)	0.030
Radiation for mBC						
None/unknown	1.00	Reference		1.00	Reference	
Any	1.00	(0.83, 1.20)	0.982	0.84	(0.67, 1.05)	0.126
Chemotherapy for mBC						
None/unknown	1.00	Reference		1.00	Reference	
Any	1.07	(0.91, 1.25)	0.390	0.93	(0.76, 1.15)	0.517
Bone metastases						
No	1.00	Reference		1.00	Reference	
Yes	1.00	(0.77, 1.30)	0.989	1.19	(0.88, 1.62)	0.247
Brain metastases						
No	1.00	Reference		1.00	Reference	
Yes	2.43	(1.60, 3.69)	< 0.001	3.53	(2.20, 5.66)	< 0.001
Liver metastases						
No	1.00	Reference		1.00	Reference	
Yes	1.92	(1.45, 2.55)	< 0.001	1.96	(2.20, 5.66)	< 0.001
Lung metastases						
No	1.00	Reference		1.00	Reference	
Yes	1.16	(0.89, 1.49)	0.270	1.26	(0.94, 1.70)	0.127

*CI* confidence interval, *DRFI* distant recurrence-free interval, *IQR* interquartile range, *mBC* metastatic breast cancer, *SD* standard deviation *SHR* subdistribution hazard ratio

Results from our sensitivity analyses examining shorter DRFI intervals were generally consistent with our findings indicating a significant trend of lower BC-specific mortality with extended DRFI (Supplemental Tables 1–3 and Supplemental Fig. 1).

## Discussion

This population-based study of women with HR + locoregional, early BC who subsequently experienced metastatic disease suggests that distant recurrence-free survival time greater than 5 years is significantly associated with ~ 30% lower risk of BC-specific mortality.

These findings and previous literature highlight the need to reduce the risk of BC recurrence early on following a first BC diagnosis. Analyses from the International BC Study Group’s clinical trials showed that estrogen receptor-positive BCs maintain a relatively high hazard of recurrence beyond 10 years post-diagnosis [3]; however, the peak hazard for recurrence occurs between 1 and 2 years after treatment. Similarly, another study of women with non-metastatic BC using the Netherlands Cancer Registry found that the highest risk for first recurrence was during the second year post-diagnosis; of these, > 70% were distant recurrences [10]. Clinical trials have evaluated extending adjuvant endocrine therapy beyond the initial five years [11]; however, extended therapy does not address the excess early risk experienced by some women with HR + BC, such as those with extensive nodal involvement (up to 5% hazards of distant recurrence annually) [12].

Building upon past studies examining the relationship between metastasis-free intervals and improved outcomes, Chang et al. followed 2,308 women with metastatic BC between 1988 and 2014 to investigate the impact of survival time between first primary BC and metastatic disease in women with both HR + and HR – disease [4]. Women with DRFI of < 5, 5–10, and > 10 years had 5 year BC-specific survival rates of 23%, 26%, and 35%, respectively. While 5 year BC-specific survival of 31% and 52% were found in HR + women with recurrent metastatic BC in this study, the poorer outcomes reported by Chang et al. likely reflect the prognosis associated with including triple negative BC. Given the diversity between BC subtypes and respective prognoses, this study focused on HR + BC, which comprises a significant proportion of recurrences and metastatic disease beyond 5 years. This study also included more years of follow-up and results adjusted for differences in treatment.

This study on DRFI covering decades of follow-up in the SEER registries may help further put into context other recent observational, registry-linked studies [13–15] following the widespread use of multi-gene prognostic assays for breast cancer recurrence [16]. Our findings showing that a higher proportion of patients with DRFI less than 5 years received chemotherapy suggest that these cases were endocrine-resistant, and whether high multi-gene recurrence scores like Oncotype DX are correlated with both lower DRFI and higher risk of distant metastasis deserves further study.

This study had several limitations. First, SEER collects data on receipt of therapy, but does not include data on treatment completion, initiation, or adherence, potentially confounding this analysis. We were also unable to distinguish between women whose diagnosis of metastatic disease following a previous diagnosis of a stage I–III BC represented a distant recurrence of their first cancer vs. a second ipsilateral or contralateral primary breast tumor presenting with de novo stage IV disease. However, fewer than 15% of patients diagnosed with a first primary breast tumor will develop a second primary breast tumor over the next 20 years [17], and only 3–5% of new BC diagnoses are de novo stage IV disease [18, 19]. Therefore, it is unlikely that more than 1–2% of this study population are women with a second primary breast tumor presenting with de novo distant metastases.

## Conclusion

Women with recurrent metastatic HR + BC and DRFI of ≥ 5 years had lower risk of BC mortality following recurrence. These findings may inform discussions between patients and clinicians surrounding both treatment approaches in early-stage BC and prognosis following metastatic recurrence. Prospective and real-world studies are needed to evaluate available adjuvant therapies and their potential to reduce the early hazards of disease recurrence in the first 5 years following early BC diagnosis.

## Supplementary Information

Below is the link to the electronic supplementary material.

Supplementary file1 (DOCX 2204 kb)

## Data Availability

Requests for datasets used and/or analyzed during the current study can be directed to the corresponding author.
