# Supplementary material for: Impact of time to distant recurrence on breast cancer-specific mortality in hormone receptor-positive breast cancer
Source: Cancer Causes Control. 2022 Feb 28;33(5):793–9. doi: 10.1007/s10552-022-01561-2 (PMC9010392; doi:10.1007/s10552-022-01561-2)

| **Supplemental Table 1.** Descriptive characteristics of women diagnosed with stages I-III hormone receptor-positive breast cancer by subsequent distant recurrence-free interval | | | | | | | | | | | | |
| --- | --- | --- | --- | --- | --- | --- | --- | --- | --- | --- | --- | --- |
|  | **All women**  **(N=1,057)** | | **DRFI <2 years**  **(n=139)** | | **DRFI 2-5 years**  **(n=230)** | | **DRFI 5-7 years**  **(n=153)** | | **DRFI 7-10 years**  **(n=241)** | | **DRFI 10+ years**  **(n=294)** | |
|  | n | (%) | n | (%) | n | (%) | n | (%) | n | (%) | n | (%) |
| **Age at Primary BC diagnosis, years** |  |  |  |  |  |  |  |  |  |  |  |  |
| Mean (SD) | 54.4 | (13.1) | 56.4 | (14.7) | 54.4 | (13.5) | 55.2 | (13.7) | 53.9 | (13.0) | 53.5 | (11.5) |
| Median (IQR) | 54 | (44-64) | 56 | (45-66) | 55 | (43-63) | 54 | (46-65) | 52 | (44-63) | 53 | (44-62) |
| <45 | 267 | (25.3) | 31 | (22.3) | 63 | (27.4) | 36 | (23.5) | 62 | (25.7) | 75 | (25.5) |
| 45-54 | 295 | (27.9) | 35 | (25.2) | 51 | (22.2) | 41 | (26.8) | 75 | (31.1) | 93 | (31.6) |
| 55-64 | 242 | (22.9) | 33 | (23.7) | 63 | (27.4) | 35 | (22.9) | 48 | (19.9) | 63 | (21.4) |
| 65-74 | 173 | (16.4) | 22 | (15.8) | 36 | (15.7) | 24 | (15.7) | 40 | (16.6) | 51 | (17.3) |
| 75+ | 80 | (7.6) | 18 | (12.9) | 17 | (7.4) | 17 | (11.1) | 16 | (6.6) | 12 | (4.1) |
| **Primary BC diagnosis year** |  |  |  |  |  |  |  |  |  |  |  |  |
| 1990-1999 | 320 | (30.3) | 27 | (19.4) | 47 | (20.4) | 22 | (14.4) | 59 | (24.5) | 165 | (56.1) |
| 2000-2009 | 624 | (59.0) | 62 | (44.6) | 135 | (58.7) | 116 | (75.8) | 182 | (75.5) | 129 | (43.9) |
| 2010-2016 | 113 | (10.7) | 50 | (36.0) | 48 | (20.9) | 15 | (9.8) | 0 | (0.0) | 0 | (0.0) |
| **Race** |  |  |  |  |  |  |  |  |  |  |  |  |
| White | 781 | (73.9) | 107 | (77.0) | 150 | (65.2) | 116 | (75.8) | 181 | (75.1) | 227 | (77.2) |
| Black | 171 | (16.2) | 23 | (16.5) | 49 | (21.3) | 23 | (15.0) | 41 | (17.0) | 35 | (11.9) |
| Other | 104 | (9.8) | 9 | (6.5) | 31 | (13.5) | 14 | (9.2) | 18 | (7.5) | 32 | (10.9) |
| **Marital status** |  |  |  |  |  |  |  |  |  |  |  |  |
| Unmarried | 442 | (41.8) | 68 | (48.9) | 100 | (43.5) | 62 | (40.5) | 100 | (41.5) | 112 | (38.1) |
| Married | 583 | (55.2) | 69 | (49.6) | 121 | (52.6) | 85 | (55.6) | 134 | (55.6) | 174 | (59.2) |
| **Stage** |  |  |  |  |  |  |  |  |  |  |  |  |
| I | 356 | (33.7) | 33 | (23.7) | 52 | (22.6) | 57 | (37.3) | 84 | (34.9) | 130 | (44.2) |
| II | 407 | (38.5) | 40 | (28.8) | 80 | (34.8) | 66 | (43.1) | 103 | (42.7) | 118 | (40.1) |
| III | 294 | (27.8) | 66 | (47.5) | 98 | (42.6) | 30 | (19.6) | 54 | (22.4) | 46 | (15.6) |
| **Grade** |  |  |  |  |  |  |  |  |  |  |  |  |
| 1 | 143 | (13.5) | 18 | (12.9) | 21 | (9.1) | 18 | (11.8) | 35 | (14.5) | 51 | (17.3) |
| 2 | 445 | (42.1) | 48 | (34.5) | 100 | (43.5) | 58 | (37.9) | 109 | (45.2) | 130 | (44.2) |
| 3 and 4 | 365 | (34.5) | 55 | (39.6) | 92 | (40.0) | 59 | (38.6) | 79 | (32.8) | 80 | (27.2) |
| Unknown | 104 |  | 18 |  | 17 |  | 18 |  | 18 |  | 33 |  |
| **Tumor size, cm** |  |  |  |  |  |  |  |  |  |  |  |  |
| <2 | 155 | (14.7) | 52 | (37.4) | 78 | (33.9) | 24 | (15.7) | 33 | (13.7) | 58 | (19.7) |
| 2-5 | 360 | (34.1) | 40 | (28.8) | 96 | (41.7) | 57 | (37.3) | 84 | (34.9) | 83 | (28.2) |
| >5 | 161 | (15.2) | 47 | (33.8) | 56 | (24.3) | 16 | (10.5) | 28 | (11.6) | 14 | (4.8) |
| **Nodal status** |  |  |  |  |  |  |  |  |  |  |  |  |
| Negative | 460 | (43.5) | 30 | (21.6) | 83 | (36.1) | 83 | (54.2) | 77 | (32.0) | 114 | (38.8) |
| Positive | 597 | (56.5) | 109 | (78.4) | 147 | (63.9) | 76 | (49.7) | 127 | (52.7) | 138 | (46.9) |
| 1 to 3 | 267 | (25.3) | 30 | (21.6) | 52 | (22.6) | 39 | (25.5) | 65 | (27.0) | 81 | (27.6) |
| 4+ | 330 | (31.2) | 79 | (56.8) | 95 | (41.3) | 37 | (24.2) | 62 | (25.7) | 57 | (19.4) |
| **Laterality of primary BC** |  |  |  |  |  |  |  |  |  |  |  |  |
| Right | 550 | (52.0) | 65 | (46.8) | 115 | (50.0) | 88 | (57.5) | 131 | (54.4) | 151 | (51.4) |
| Left | 507 | (48.0) | 74 | (53.2) | 115 | (50.0) | 65 | (42.5) | 110 | (45.6) | 143 | (48.6) |
| **Surgery** |  |  |  |  |  |  |  |  |  |  |  |  |
| Breast-conserving | 565 | (53.5) | 42 | (30.2) | 99 | (43.0) | 85 | (55.6) | 144 | (59.8) | 195 | (66.3) |
| Mastectomy | 459 | (43.4) | 80 | (57.6) | 125 | (54.3) | 65 | (42.5) | 92 | (38.2) | 97 | (33.0) |
| Unknown type | 33 |  | 17 |  | 6 |  | 3 |  | 5 |  | 2 |  |
| **Radiation** |  |  |  |  |  |  |  |  |  |  |  |  |
| None/unknown | 476 | (45.0) | 78 | (56.1) | 100 | (43.5) | 73 | (47.7) | 98 | (40.7) | 127 | (43.2) |
| Any | 557 | (52.7) | 58 | (41.7) | 122 | (53.0) | 77 | (50.3) | 139 | (57.7) | 161 | (54.8) |
| **Chemotherapy** |  |  |  |  |  |  |  |  |  |  |  |  |
| None/unknown | 526 | (49.8) | 68 | (48.9) | 92 | (40.0) | 80 | (52.3) | 119 | (49.4) | 167 | (56.8) |
| Any | 531 | (50.2) | 71 | (51.1) | 138 | (60.0) | 73 | (47.7) | 122 | (50.6) | 127 | (43.2) |

| **Supplemental Table 2.** Descriptive characteristics of women diagnosed with stage IV hormone receptor-positive breast cancer by preceding distant recurrence-free interval | | | | | | | | | | | | |
| --- | --- | --- | --- | --- | --- | --- | --- | --- | --- | --- | --- | --- |
|  | **All women**  **(N=1,057)** | | **DRFI <2 years**  **(n=139)** | | **DRFI 2-5 years**  **(n=230)** | | **DRFI 5-7 years**  **(n=153)** | | **DRFI 7-10 years**  **(n=241)** | | **DRFI 10+ years**  **(n=294)** | |
|  | n | (%) | n | (%) | n | (%) | n | (%) | n | (%) | n | (%) |
| **Age at MBC diagnosis, years** |  |  |  |  |  |  |  |  |  |  |  |  |
| Mean (SD) | 61.8 | (13.3) | 57.2 | (14.6) | 57.7 | (13.4) | 61.2 | (13.6) | 62.1 | (12.7) | 67.4 | (11.1) |
| Median (IQR) | 62 | (52-72) | 57 | (46-66) | 58 | (46-67) | 60 | (52-71) | 61 | (52-72) | 66 | (59-76) |
| <45 | 115 | (10.9) | 30 | (21.6) | 49 | (21.3) | 19 | (12.4) | 15 | (6.2) | 2 | (0.7) |
| 45-54 | 206 | (19.5) | 32 | (23.0) | 42 | (18.3) | 31 | (20.3) | 59 | (24.5) | 42 | (14.3) |
| 55-64 | 296 | (28.0) | 33 | (23.7) | 71 | (30.9) | 43 | (28.1) | 71 | (29.5) | 78 | (26.5) |
| 65-74 | 223 | (21.1) | 23 | (16.5) | 36 | (15.7) | 31 | (20.3) | 46 | (19.1) | 87 | (29.6) |
| 75+ | 217 | (20.5) | 21 | (15.1) | 32 | (13.9) | 29 | (19.0) | 50 | (20.7) | 85 | (28.9) |
| **MBC diagnosis year** |  |  |  |  |  |  |  |  |  |  |  |  |
| 1990-1999 | 67 | (6.3) | 25 | (18.0) | 28 | (12.2) | 10 | (6.5) | 4 | (1.7) | 0 | (0.0) |
| 2000-2009 | 341 | (32.3) | 60 | (43.2) | 101 | (43.9) | 40 | (26.1) | 79 | (32.8) | 61 | (20.7) |
| 2010-2016 | 649 | (61.4) | 54 | (38.8) | 101 | (43.9) | 103 | (67.3) | 158 | (65.6) | 233 | (79.3) |
| **Radiation** |  |  |  |  |  |  |  |  |  |  |  |  |
| None/unknown | 476 | (45.0) | 103 | (74.1) | 177 | (77.0) | 73 | (47.7) | 98 | (40.7) | 127 | (43.2) |
| Any | 557 | (52.7) | 33 | (23.7) | 51 | (22.2) | 77 | (50.3) | 139 | (57.7) | 161 | (54.8) |
| **Chemotherapy** |  |  |  |  |  |  |  |  |  |  |  |  |
| None/unknown | 526 | (49.8) | 68 | (48.9) | 112 | (48.7) | 80 | (52.3) | 119 | (49.4) | 167 | (56.8) |
| Any | 531 | (50.2) | 74 | (53.2) | 118 | (51.3) | 73 | (47.7) | 122 | (50.6) | 127 | (43.2) |
| ***Metastatic site involvement (2010+)*** |  |  |  |  |  |  |  |  |  |  |  |  |
| **Bone** |  |  |  |  |  |  |  |  |  |  |  |  |
| No | 209 | (19.8) | 14 | (10.1) | 34 | (14.8) | 30 | (19.6) | 47 | (19.5) | 84 | (28.6) |
| Yes | 420 | (39.7) | 25 | (18.0) | 63 | (27.4) | 71 | (46.4) | 106 | (44.0) | 145 | (49.3) |
| Unknown | 20 |  | 5 |  | 4 |  | 2 |  | 5 |  | 4 |  |
| Not 2010+ | 408 |  | 85 |  | 129 |  | 50 |  | 83 |  | 61 |  |
| **Brain** |  |  |  |  |  |  |  |  |  |  |  |  |
| No | 569 | (53.8) | 45 | (32.4) | 84 | (36.5) | 85 | (55.6) | 142 | (58.9) | 213 | (72.4) |
| Yes | 47 | (4.4) | 4 | (2.9) | 11 | (4.8) | 11 | (7.2) | 9 | (3.7) | 12 | (4.1) |
| Unknown | 33 |  | 5 |  | 6 |  | 7 |  | 7 |  | 8 |  |
| Not 2010+ | 408 |  | 85 |  | 129 |  | 50 |  | 83 |  | 61 |  |
| **Liver** |  |  |  |  |  |  |  |  |  |  |  |  |
| No | 492 | (46.5) | 39 | (28.1) | 65 | (28.3) | 77 | (50.3) | 123 | (51.0) | 188 | (63.9) |
| Yes | 127 | (12.0) | 10 | (7.2) | 31 | (13.5) | 22 | (14.4) | 29 | (12.0) | 35 | (11.9) |
| Unknown | 30 |  | 5 |  | 5 |  | 4 |  | 6 |  | 10 |  |
| Not 2010+ | 408 |  | 85 |  | 129 |  | 50 |  | 83 |  | 61 |  |
| **Lung** |  |  |  |  |  |  |  |  |  |  |  |  |
| No | 424 | (40.1) | 38 | (27.3) | 73 | (31.7) | 72 | (47.1) | 94 | (39.0) | 147 | (50.0) |
| Yes | 192 | (18.2) | 12 | (8.6) | 22 | (9.6) | 24 | (15.7) | 57 | (23.7) | 77 | (26.2) |
| Unknown | 33 |  | 4 |  | 6 |  | 7 |  | 7 |  | 9 |  |
| Not 2010+ | 408 |  | 85 |  | 129 |  | 50 |  | 83 |  | 61 |  |

| **Supplemental Table 3.** Results from Fine and Grey competing risks regression models reporting subdistribution hazard ratios (SHR) and robust 95% confidence intervals (CI) for risk of breast cancer-specific mortality following metastatic progression of hormone receptor-positive breast cancer | | | | | | |
| --- | --- | --- | --- | --- | --- | --- |
|  | **Crude** |  |  | **Adjusted** |  |  |
|  | **SHR** | **Robust 95% CI** | **P** | **SHR** | **Robust 95% CI** | **P** |
| **Distant recurrence-free interval** |  |  |  |  |  |  |
| <2 years | 1.00 | Reference |  | 1.00 | Reference |  |
| 2-5 years | 1.02 | (0.84, 1.25) | 0.796 | 0.99 | (0.78, 1.26) | 0.952 |
| 5-7 years | 0.84 | (0.67, 1.05) | 0.128 | 0.92 | (0.69, 1.22) | 0.550 |
| 7-10 years | 0.60 | (0.48, 0.74) | <0.001 | 0.67 | (0.51, 0.87) | 0.003 |
| 10+ years | 0.50 | (0.40, 0.62) | <0.001 | 0.51 | (0.39, 0.68) | <0.001 |
| **Age at MBC diagnosis** |  |  |  |  |  |  |
| <45 | 1.00 | Reference |  | 1.00 | Reference |  |
| 45-54 | 0.77 | (0.64, 0.94) | 0.010 | 1.00 | (0.80, 1.25) | 0.989 |
| 55-64 | 0.77 | (0.64, 0.93) | 0.006 | 1.03 | (0.84, 1.27) | 0.756 |
| 65-74 | 0.67 | (0.55, 0.83) | <0.001 | 0.85 | (0.65, 1.10) | 0.211 |
| 75+ | 0.84 | (0.68, 1.04) | 0.107 | 1.30 | (0.99, 1.71) | 0.062 |
| **MBC diagnosis year** |  |  |  |  |  |  |
| 1990-1999 | 1.00 | Reference |  | 1.00 | Reference |  |
| 2000-2009 | 0.72 | (0.59, 0.88) | 0.001 | 0.88 | (0.68, 1.12) | 0.287 |
| 2010-2016 | 0.56 | (0.45, 0.68) | <0.001 | 0.53 | (0.38, 0.75) | <0.001 |
| **Race** |  |  |  |  |  |  |
| White | 1.00 | Reference |  | 1.00 | Reference |  |
| Black | 1.29 | (1.10, 1.53) | 0.002 | 1.28 | (1.05, 1.55) | 0.014 |
| Other | 0.99 | (0.79, 1.25) | 0.954 | 1.20 | (0.92, 1.57) | 0.171 |
| **Grade** |  |  |  |  |  |  |
| 1 | 1.00 | Reference |  | 1.00 | Reference |  |
| 2 | 1.39 | (1.06, 1.83) | 0.019 | 1.32 | (0.98, 1.78) | 0.065 |
| 3 and 4 | 2.01 | (1.53, 2.64) | <0.001 | 1.74 | (1.29, 2.34) | <0.001 |
| **Radiation for MBC** |  |  |  |  |  |  |
| None/unknown | 1.00 | Reference |  | 1.00 | Reference |  |
| Any | 1.00 | (0.87, 1.16) | 0.963 | 0.89 | (0.75, 1.06) | 0.199 |
| **Chemotherapy for MBC** |  |  |  |  |  |  |
| None/unknown | 1.00 | Reference |  | 1.00 | Reference |  |
| Any | 1.16 | (1.02, 1.31) | 0.021 | 0.96 | (0.82, 1.13) | 0.654 |
| **Bone metastases** |  |  |  |  |  |  |
| No | 1.00 | Reference |  | 1.00 | Reference |  |
| Yes | 0.98 | (0.79, 1.22) | 0.853 | 1.16 | (0.90, 1.50) | 0.261 |
| **Brain metastases** |  |  |  |  |  |  |
| No | 1.00 | Reference |  | 1.00 | Reference |  |
| Yes | 2.34 | (1.66, 3.30) | <0.001 | 2.54 | (1.66, 3.89) | <0.001 |
| **Liver metastases** |  |  |  |  |  |  |
| No | 1.00 | Reference |  | 1.00 | Reference |  |
| Yes | 1.76 | (1.38, 2.26) | <0.001 | 1.46 | (1.07, 2.00) | 0.017 |
| **Lung metastases** |  |  |  |  |  |  |
| No | 1.00 | Reference |  | 1.00 | Reference |  |
| Yes | 1.14 | (0.93, 1.42) | 0.202 | 1.26 | (0.98, 1.62) | 0.067 |

**Supplemental Figure 1.** Kaplan Meier survivor functions for breast cancer-specific mortality by distant recurrence-free interval


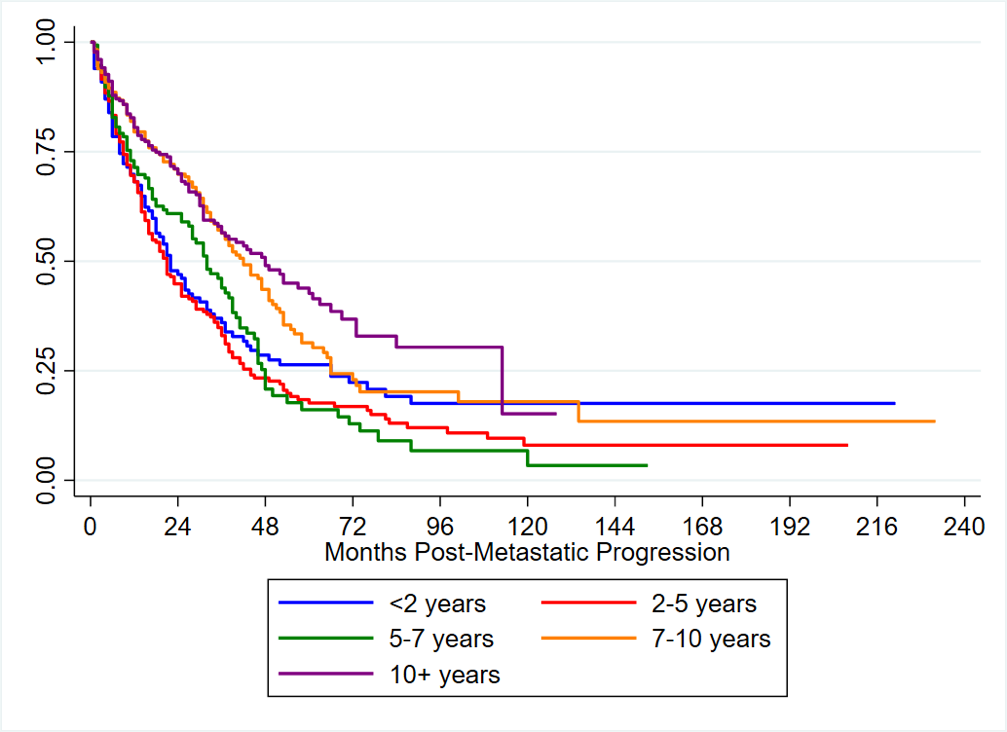

Supplement: Supplementary file 1 — Supplementary file1 (DOCX 2204 kb) [file 10552_2022_1561_MOESM1_ESM.docx]
